# Experimental and Theoretical Insights into the Role of Iron in the Rapidly Fabricated Ni/Fe Electrodes for the Oxygen Evolution Reaction

**DOI:** 10.1002/cssc.202500281

**Published:** 2025-06-24

**Authors:** Yue Wang, Gustavo T. Feliciano, Ashwani Kumar, Alexander A. Auer, Harun Tüysüz

**Affiliations:** ^1^ Department of Heterogeneous Catalysis Max‐Planck‐Institut für Kohlenforschung Kaiser‐Wilhelm‐Platz 1 D‐45470 Mülheim an der Ruhr Germany; ^2^ Department of Molecular Theory and Spectroscopy Max‐Planck‐Institut für Kohlenforschung Kaiser‐Wilhelm‐Platz 1 D‐45470 Mülheim an der Ruhr Germany; ^3^ Catalysis and Energy Materials Group IMDEA Materials Institute Calle Eric Kandel 2 28906 Getafe Madrid Spain

**Keywords:** density functional theory, electrocatalysts, electronic structure calculations, Fe incorporation, nickel oxy‐hydroxide, oxygen evolution reaction, water splitting

## Abstract

The development of low‐cost electrocatalysts for the oxygen evolution reaction (OER) of water electrolysis is crucial for large‐scale green hydrogen production. NiFe‐based electrocatalysts have garnered significant attention due to their high OER activity; however, the need for a rapid and efficient electrode fabrication method and a clear understanding of the role of Fe in enhancing OER activity remains unresolved. Herein, a highly active NiFe‐based OER electrocatalyst self‐supported on carbon fiber paper (CFP) is developed using a versatile and rapid thermal shock method, requiring only 30 s of heat treatment. The as‐prepared Fe_1_Ni_1_/CFP shows a current density of 493 mA/cm^2^ at 1.7 V vs reversible hydrogen electrode (RHE) and a low overpotential of 247 mV at 10 mA/cm^2^, with excellent long‐term durability in alkaline conditions. In situ Raman spectroscopy, pH‐dependance activity test, and electronic structure calculations reveal that Fe not only promotes the oxidation of adjacent Ni but also accelerates the deprotonation of adsorbed –OH groups and stabilizes oxo‐intermediates, thus, displaying both direct and indirect effects and enhancing the overall OER performance. This article provides a foundation for developing cost‐effective electrocatalysts for hydrogen production and other sustainable energy applications while enhancing the understanding of the role of Fe in NiO catalysts.

## Graphical abstract

A series of highly active NiFe‐based oxygen evolution reaction electrocatalysts self‐supported on carbon fiber paper is prepared using a versatile and rapid thermal shock method, requiring only 30 s of heat treatment. The optimized electrocatalyst (Fe_1_Ni_1_) exhibits excellent activity and long‐term stability in alkaline conditions,    requiring a low overpotential of only 247 mV at 10 mA cm^‐^^2^.

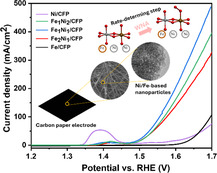

## 1 Introduction

Hydrogen, characterized by its high energy storage density and zero carbon emissions, is considered one of the most promising alternatives to fossil fuels and future energy vectors.^[^ ^1^ ^]^ Green hydrogen is produced through water electrolysis with electricity generated from renewable energy sources without CO_2_ emission and environmental drawbacks.^[^ ^2^ ^]^ Water electrolysis consists of two half‐reactions, the hydrogen evolution reaction (HER) and the oxygen evolution reaction (OER). In particular, the OER, which is characterized by a four‐electron transfer process, is inherently slower than the HER, which involves only a two‐electron transfer.^[3a]^ Therefore, developing efficient OER catalysts remains critical to minimizing losses in green hydrogen production and making the process more cost‐effective.

Benchmark OER catalysts, such as IrO_2_ and RuO_2_, still face limitations in practical industrial applications due to their high cost and scarcity. Recent investigations have identified low‐cost earth‐abundant transition metal (TM: Co, Ni, Fe)‐based catalysts as promising alternatives due to their widespread availability and excellent OER performance.^[^ ^3^ ^]^ TM oxides, particularly perovskites^[^ ^4^ ^]^ and spinel oxides,^[^ ^5^ ^]^ have gained attention for their simple synthesis methods and tunable physicochemical properties. Upon surface reconstruction in alkaline electrolytes, oxides transform into (oxy)hydroxides, which are reported as the real active species in OER.^[^ ^6^ ^]^ Consequently, research has focused on TM (oxy)hydroxide catalysts, particularly layered double hydroxides, which have exhibited enhanced catalytic activity.^[^ ^7^ ^]^ Utilizing the synergistic effects of various TMs to modulate the electronic structure has proven to enhance OER efficiency further. For instance, CoNi mixed oxides have shown superior activity compared to individual NiO or Co_3_O_4_ counterparts, and Fe plays an important role in enhancing the activity of Co‐ or Ni‐based catalysts.^[^ ^8^ ^]^ Despite the increasing interest in NiFe‐based catalysts due to their high intrinsic activity, the mechanistic understanding of OER catalysis, especially the role of Fe, remains ambiguous, with multiple hypotheses proposed. Li et al. suggested that Fe^3+^ behaves as a Lewis acid to promote the formation of OER‐active Ni^4+^ species.^[^ ^9^ ^]^ While Friebel et al. found that the Fe cations are the active sites in Ni_1–*x*_Fe_*x*_OOH. The electronic properties of Fe cations are changed when they are incorporated into NiOOH, making them OER‐active.^[^ ^10^ ^]^ Additionally, the synergy between Ni and Fe is also reported, with Fe facilitating O radical delivery and Ni facilitating O–O coupling, collectively contributing to activity enhancement.^[^ ^11^ ^]^

Among different physicochemical properties, crystal structure and degree of crystallinity are key factors that significantly influence OER performance. Catalysts with disordered structures or defects are more likely to form active intermediates. Indra et al. reported higher OER performance of amorphous cobalt iron oxide compared to its highly crystalline counterpart, albeit suffering from rapid degradation.^[^ ^12^ ^]^ Controlling the degree of crystallinity of Co oxide by precipitation at room temperature followed by various calcination steps at different temperatures has been shown to optimize OER performance. Partially crystalline structures show superior activity compared to both crystalline and amorphous structures with excellent stability.^[^ ^13^ ^]^

Furthermore, the fabrication of the working electrode also plays a crucial role in reducing the overall cost of electrolysis. Typically, active catalyst materials are synthesized as powders, and then, the corresponding ink solution is coated onto electrode substrates with an electrically conductive polymer as a binder. However, this process is time‐consuming and influenced by various factors, such as ink solution homogeneity, binder quantity, coating method, and drying conditions. Therefore, the development of rapid and straightforward electrode fabrication methods is imperative to meet the demands of practical applications, ensuring scalability, cost‐effectiveness, and efficiency. Such methods play a critical role in bridging the gap between research development and real‐world implementation, enabling the widespread adoption of advanced electrocatalysts.^[3b]^ The rapid and facile thermal shock synthesis method allows precise adjustment of both temperature and duration, facilitating the controlled preparation of target materials with tailored crystallinity and structural properties. Additionally, the short duration of thermal shock limits migration and sintering of nanoparticles.

Enhancing catalyst activity can be achieved more effectively by uncovering key structure–activity relationships and gaining mechanistic insights through the application of advanced electronic structure methodologies. Wang et al.^[^ ^14^ ^]^ utilized density functional theory (DFT) simulations to demonstrate that the OER mechanism in Ni, Fe, and Co bifunctional metal oxides is highly potential‐dependent and varies across these materials. Ou et al.^[^ ^15^ ^]^ highlighted the cooperative role of Fe atoms, emphasizing that closely positioned oxo groups generated at very low overpotentials significantly enhance catalytic activity. Xiao et al.^[^ ^16^ ^]^ proposed a synergistic interaction between Ni and Fe during O—O bond formation, with Ni playing a more intricate role in the process. Li et al.^[^ ^17^ ^]^ demonstrated that Fe^3+^ cations, acting as Lewis acid dopants, indirectly influence Ni by stabilizing Ni^4+^ species in nickel oxide films, thereby enhancing OER activity. Bhattacharyya et al.^[^ ^18^ ^]^ provided a comprehensive study of pH‐ and potential‐dependent interface structures in Co oxides, illustrating how Fe incorporation into the CoOx lattice alters the energetics of rate‐determining steps. The authors also stress the importance of the explicit incorporation of pH and potential in the simulation approach.^[^ ^19^ ^]^

In this article, we utilized a rapid thermal shock method to fabricate NiFe‐based electrocatalysts with varying Ni/Fe ratios, self‐supported on carbon fiber paper (CFP), for alkaline OER. The swift thermal treatment produced catalysts with nanoparticle morphology, low crystallinity, and high oxo content, resulting in exceptional OER performance. In situ electrochemical Raman spectroscopy revealed that Fe promoted the rapid formation, high population, and stability of OER‐active oxyhydroxide and superoxide species, particularly in Fe_1_Ni_1_/CFP, which demonstrated the highest activity. Electronic structure calculations on nickel oxyhydroxide models, with and without Fe incorporation, under constant pH and potential conditions, provided further insights into OER mechanisms. Reaction profiles identified rate‐limiting steps and clarified the role of Fe in enhancing catalytic performance.

## 2 Results and Discussion

To explore how Fe incorporation enhances the OER activity of NiFe‐based electrocatalysts, we synthesized self‐supported NiFe‐based electrocatalysts on a CFP substrate using a rapid thermal shock process. For detailed experimental procedures, see the Experimental Section. A series of samples were prepared by varying the ratio of Fe and Ni precursors during the synthesis, denoted as Fe/CFP, Ni/CFP, and Fe_*x*_Ni_*y*_/CFP (*x*:*y* = 1:1, 2:1, and 1:2). All prepared electrodes were subjected to thermal treatment at 500 °C for 30 s, unless stated otherwise.

**Figure 1:**
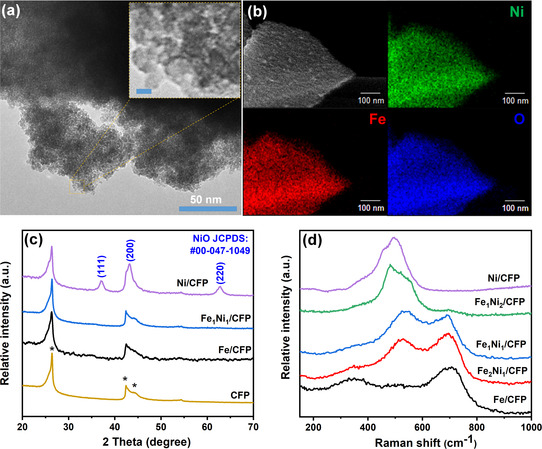
a) TEM image of Fe_1_Ni_1_/CFP, insert figure is the HR‐TEM with 2 nm scale bar. b) High‐angle annular dark‐field scanning transmission electron microscopy (HAADF‐STEM) image of Fe_1_Ni_1_/CFP with corresponding elemental mapping. c) XRD patterns and d) Raman spectra of all electrodes.

High‐resolution transmission electron microscopy (HR‐TEM) images of the materials on selected electrodes are presented in **Figure** **1**a and Figure S1 (Supporting Information). In Fe/CFP and Fe_1_Ni_1_/CFP electrodes, the metal species appear as nanoparticles with an approximate size of 10 nm without any obvious degree of crystallinity. Conversely, the Ni/CFP electrode displays aggregated small nanoparticles with a crystalline phase (Figure S1a, Supporting Information). The lattice fringes observed in Figure S1a (Supporting Information) indicate an interplanar distance of 0.24 nm, corresponding to the (111) lattice plane in NiO. Elemental mapping of the selected Fe_1_Ni_1_/CFP (Figure 1b) reveals the homogeneous distribution of Fe and Ni across the sample. Scanning electron microscopy images with energy‐dispersive X‐ray spectroscopy (EDX) analysis of bimetallic electrodes (Figure S2, Supporting Information) reveal that the metal‐based catalysts are small particles located in the gaps between carbon fibers. This might facilitate good electrical conductivity and protection against catalyst dissolution in the electrolyte during OER. The Fe/Ni ratios in these electrodes are confirmed to align with theoretical values, as indicated in the table in Figure S2d (Supporting Information).

The X‐ray diffraction (XRD) patterns of selected electrodes were obtained with the transmission mode, as shown in Figure 1c. No diffraction peaks are observed in Fe/CFP and Fe_1_Ni_1_/CFP, except for those arising from CFP. Conversely, Ni/CFP exhibits three diffraction peaks at 37°, 43°, and 63°, matching the standard NiO (#00‐047‐1049), indicating the presence of crystalline NiO domains in Ni/CFP and amorphous phases in Fe/CFP and Fe_1_Ni_1_/CFP. This observation is consistent with TEM analyses. Further investigation of the thermal shock and heating effects was conducted by adjusting the heating duration of the electrodes. The obtained XRD patterns of the samples are shown in Figure S3 (Supporting Information). Fe/CFP exhibits a crystalline Fe_2_O_3_ hematite phase after 1 min of thermal treatment, suggesting that the Fe species in Fe/CFP are compounds resulting from incomplete thermal decomposition of Fe(NO_3_)_3_·9H_2_O precursor. The intensity and sharpness of NiO diffraction peaks in Ni/CFP increase with longer thermal treatment times of 1 and 3 min due to enhanced crystallinity and formation of larger particles. Fe_1_Ni_1_/CFP exhibited only two small humps around 37° and 62° after a longer thermal treatment of 3 min, indicating limited structural changes.

Raman spectra of all prepared electrodes with thermal treatment times of 30 s are shown in Figure 1d. Fe/CFP displays two clear bands around 350 and 700 cm^−1^ and a small hump around 500 cm^−1^, consistent with the Raman spectrum of mostly amorphous hydrous iron (oxy)hydroxide.^[^ ^20^ ^]^ Figure S4b (Supporting Information) shows that the iron (oxy)hydroxide in Fe/CFP turns into Fe_2_O_3_ hematite when a higher laser intensity is applied.^[20c]^ This is in good agreement with the change of the XRD pattern of Fe/CFP upon longer thermal treatment time in the oven. The spectrum of Ni/CFP with a band at ≈500 cm^−1^ and a shoulder at ≈420 cm^−1^ further supports that the Ni species in Ni/CFP is NiO.^[^ ^21^ ^]^ The Fe_1_Ni_2_/CFP shows a band at ≈470 cm^−1^ and a shoulder at ≈550 cm^−1^, which may be a contribution from disordered Ni hydroxide with Fe incorporation.^[^ ^22^ ^]^ Fe_1_Ni_1_/CFP and Fe_2_Ni_1_/CFP have similar spectra with two broad bands at ≈530 and ≈700 cm^−1^, which are related to the FeNi hydroxide.^[^ ^22^ ^]^

X‐ray photoelectron spectroscopy (XPS) was employed to analyze the surface oxidation states of samples, as shown in Figure S5 (Supporting Information). Fe/CFP exhibits Fe 2p_1/2_ and Fe 2p_3/2_ peaks centered at 724.5 and 710.5 eV, respectively, corresponding to Fe^3+^ in iron (oxy)hydroxide.^[^ ^23^ ^]^ These peaks are shifted to higher binding energy in Fe_1_Ni_1_/CFP, indicating a higher average oxidation state of Fe due to interaction with Ni. The XPS spectrum of Ni/CFP (Figure S5b, Supporting Information) displays a Ni 2p_3/2_ peak at 655.5 eV and Ni 2p_1/2_ peak at 673.1 eV, suggesting a mixture of Ni^2+^ and Ni^3+^ in possible species of NiO and nickel hydroxide.^[^ ^24^ ^]^ These peaks exhibit the same position but appear broader in Fe_1_Ni_1_/CFP. The O 1s spectra (Figure S5c, Supporting Information) reveal a peak at 529.5 eV and a shoulder at 531.3 eV for Fe/CFP, hinting at the existence of Fe—O and Fe—OH bonds.^[23a]^ Fe_1_Ni_1_/CFP exhibits two peaks at similar positions, with the higher binding energy peak having greater intensity due to higher –OH content. Ni/CFP displays a broad O 1s peak in the range of 529–534 eV, attributed to Ni oxide, hydroxide, and oxyhydroxide species.^[^ ^24^ ^]^ The detailed characterization results indicate that a short thermal treatment of 30 s results in amorphous iron (oxy)hydroxide nanoparticles supported on CFP, while Ni‐rich samples tend to go through a rapid crystallization process by forming their crystalline oxide counterparts.

After detailed structural characterization, the OER performance of the prepared electrodes was evaluated using linear sweep voltammetry (LSV) curves following stabilization with 100 cyclic voltammetry (CV) cycles. Initially, we examined the impact of heating time and the degree of crystallization on selected electrodes, as shown in Figure S6 (Supporting Information). A prolonged thermal treatment led to a decline in OER activity for Fe/CFP, Ni/CFP, and Fe_1_Ni_1_/CFP electrodes, likely due to increased crystallinity, particle sintering, and potential damage to the CFP when heated in air. Therefore, we optimized and maintained a heating time of 30 s for all further investigations in this study.

**Figure 2:**
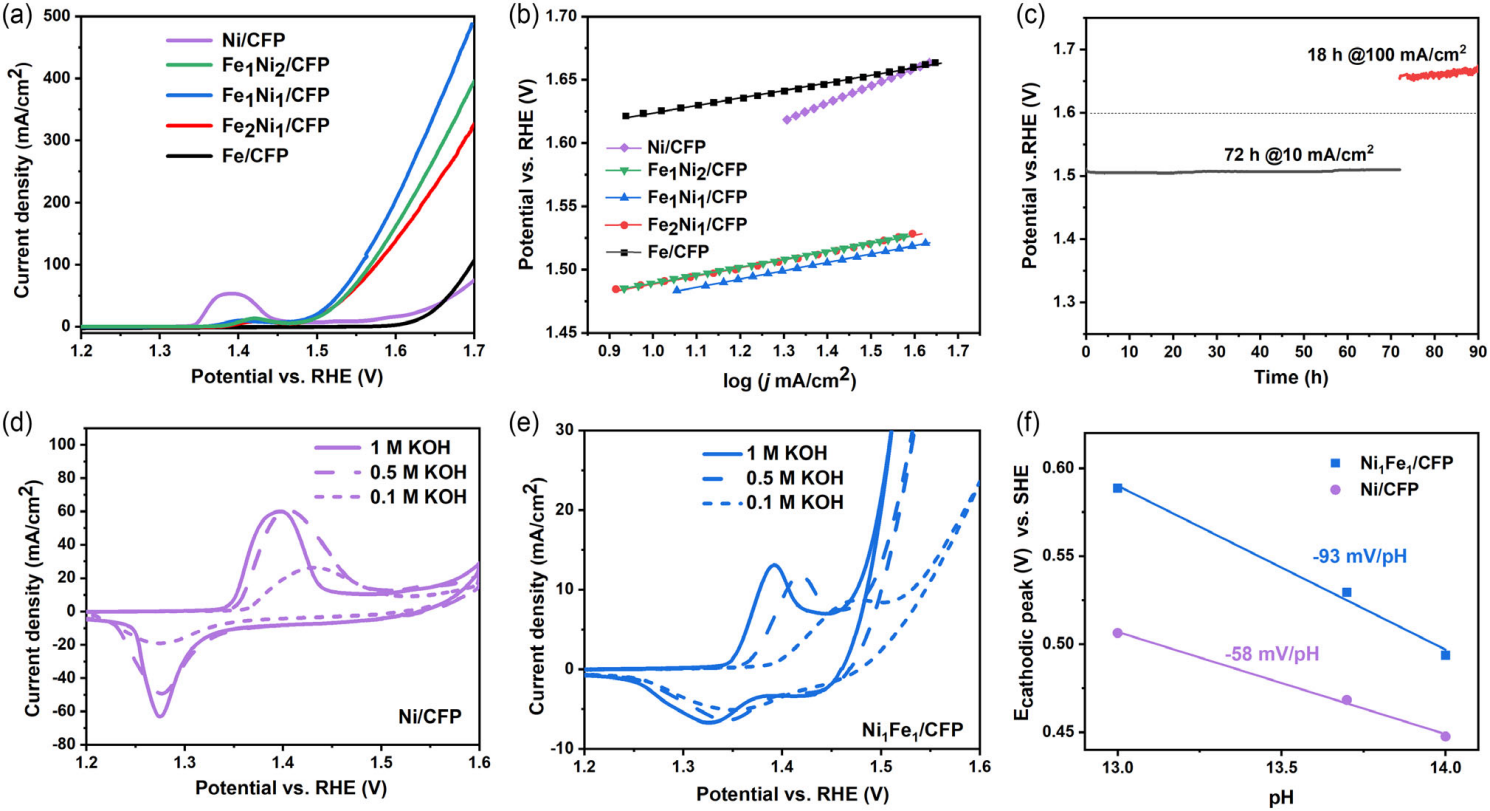
a) LSV curves and b) calculated Tafel slopes of all electrodes after 100 CV in 1 M KOH. c) CP measurement of Fe_1_Ni_1_/CFP at 10 mA cm^−2^ for 72 h, followed by 100 mA cm^−2^ for 18 h. The 100^th^ CV curves in electrolyte with various pH of d) Ni/CFP and e) Fe_1_Ni_1_/CFP. f) The calculated Pourbaix slopes of Ni/CFP and Fe_1_Ni_1_/CFP.

The LSV curves of all electrodes with thermal shock treatment of 30 s are shown in **Figure** **2**a. Among all samples, Fe_1_Ni_1_/CFP exhibits the highest activity, achieving a high current density (*j*_*@1.7 V*_) of 493 mA cm^−2^ at 1.7 Vreversible hydrogen electrode (RHE) and a low overpotential (*η*_10_) of 247 mV to reach a current density of 10 mA cm^−2^. Fe_1_Ni_2_/CFP demonstrates a higher *j*_*@1.7 V*_ (394 mA cm^−2^) but similar *η*_10_ (260 mV) compared to Fe_2_Ni_1_/CFP (*j*_*@1.7 V*_ = 328 mA cm^−2^). The Fe/CFP delivers a *j*_*@1.7 V*_ of 108 mA cm^−2^ and *η*_10_ of 394 mV, comparable to the activity of mesostructured Co_3_O_4,_^[^ ^25^ ^]^ while the Ni/CFP exhibits the lowest OER activity with a *j*_*@1.7 V*_ of 75 mA cm^−2^ and *η*_10_ of 333 mV. Tafel analysis (Figure 2b) reveals similar Tafel slopes around 63–65 mV dec^−1^ for electrodes with mixtures of Fe and Ni, indicating a consistent kinetic pathway. This value aligns well with reported Tafel slopes of NiFe hydroxide.^[^ ^26^ ^]^ Fe/CFP exhibits the lowest Tafel slope of 60 mV dec^−1^ due to the high intrinsic activity of Fe,^[^ ^27^ ^]^ while Ni/CFP displays the most sluggish kinetic with a high Tafel slope of 147 mV dec^−1^, consistent with the previous finding.^[26a]^ A long‐term stability test was performed on the most active electrode, Fe_1_Ni_1_/CFP, using chronopotentiometry (CP) by maintaining a current density of 10 mA cm^−^^2^ for 72 h, followed by 100 mA cm^−^^2^ for an additional 18 h. As shown in Figure 2c, the CP curves exhibit excellent stability, with a consistent potential over the extended period. Additionally, the LSV curves before and after the CP test (Figure S7, Supporting Information) reveal no signs of activity degradation, confirming the electrode's durability over the 90 hour stability test. The activity comparison of Ni_1_Fe_1_/CFP with other reported NiFe‐based OER catalysts is presented in Table S1 (Supporting Information), highlighting the superior activity of the prepared electrocatalyst.

To uncover the possible origin of the exceptional OER performance of Fe_1_Ni_1_/CFP in comparison to pristine Fe and Ni electrodes, and to understand the effective role of Fe in enhancing the OER performance of the hybrid Fe_1_Ni_1_/CFP, we conducted pH‐dependent activity tests, in situ Raman analysis, and a computational study. First, the pH‐dependance test was conducted for Ni/CFP and Fe_1_Ni_1_/CFP electrodes to gain insights into the reaction mechanism. The CV curves were collected after activation with 100 CV cycles in KOH electrolyte with different concentrations of 1, 0.5, and 0.1 M. As seen in Figure 2d–e, the cathodic peaks corresponding to the reduction of Ni^3+/^Ni^2+^ are observed at ≈1.27 V_RHE_ in Ni/CFP and ≈1.33–1.38 V_RHE_ in Fe_1_Ni_1_/CFP. By converting these potentials to the scale of standard hydrogen electrode and plotting them against the pH value of the electrolyte, the Pourbaix slopes were obtained (Figure 2f). For the Ni/CFP electrode, the peak shift with respect to pH is calculated to be –58 mV/pH, indicating Nernstian behavior,^[^ ^28^ ^]^ a result of coupled proton–electron transfer.^[^ ^29^ ^]^ Conversely, for Fe_1_Ni_1_/CFP, the calculated slope is –93 mV/pH, indicative of two‐proton‐one‐electron transfer. This decoupled proton–electron transfer leads to the formation of negatively charged oxygen, which is crucial for the generation of active oxygen or superoxide species (M—O—O and M—O—O—M; where M represents the metal site).^[^ ^30^ ^]^

**Figure 3:**
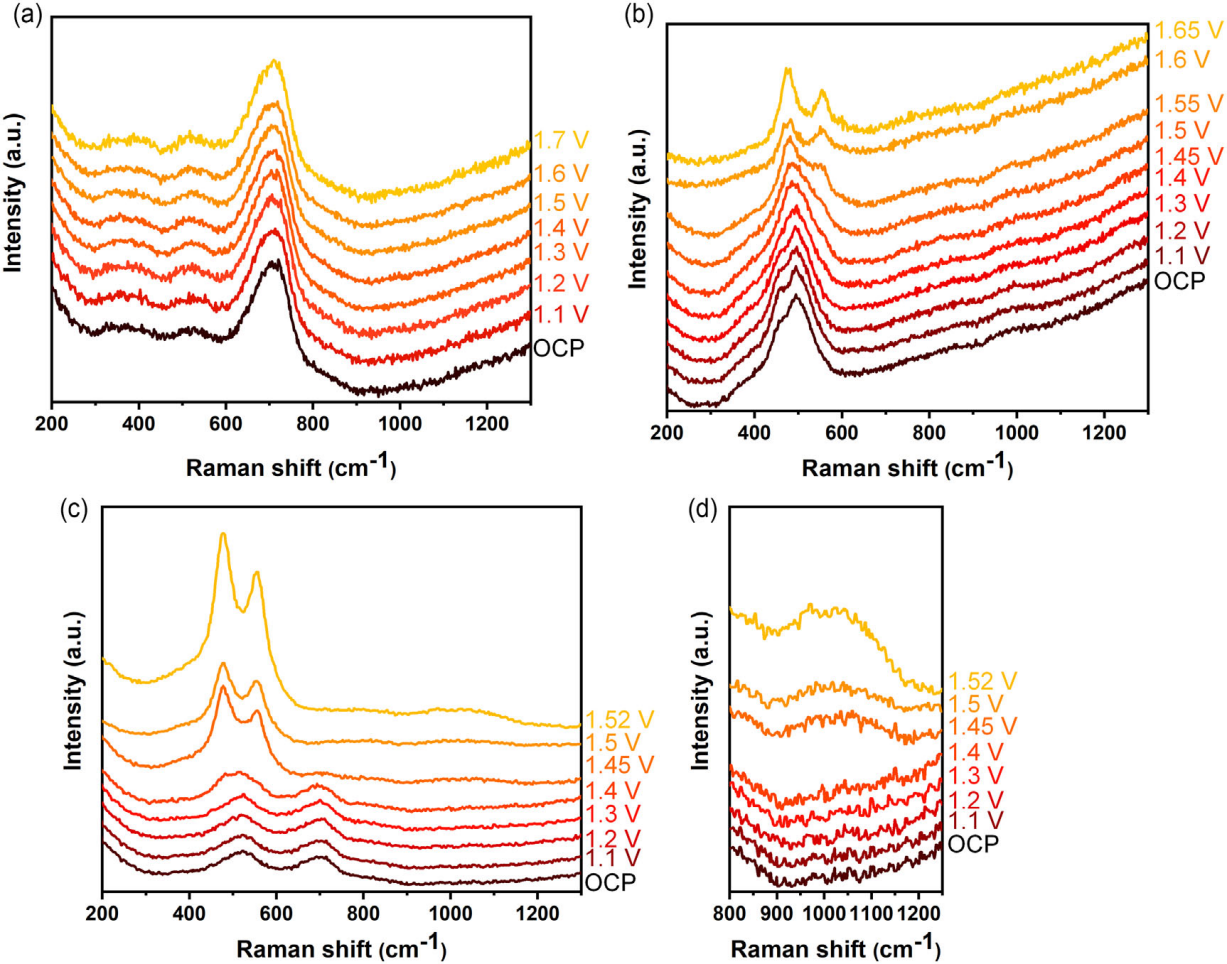
In situ Raman spectra of a) Fe/CFP, b) Ni/CFP, and c,d) Fe_1_Ni_1_/CFP in 0.1 M KOH. The OCP indicates the spectra collected after 30 min immersion and at open circuit potential. The potential endings for Ni‐containing catalysts are lower because of the bubble formation with active catalysts.

As alteration and regeneration of electrocatalysts are key factors toward large‐scale applications, the alteration of selected catalysts under working conditions was further monitored using in situ Raman microscopy coupled with a homemade electrochemical cell. The collected spectra are presented in **Figure** **3**. Upon immersion of Fe/CFP in the electrolyte for 30 min, the intensity of the hump at ≈500 cm^−1^ decreases, while the one at ≈700 cm^−1^ becomes higher and more symmetric, compared to the dried and as‐prepared Fe/CFP (Figure 3a). This change is attributed to the solvation effect of iron (oxy)hydroxide.^[20a]^ When a potential is applied, Fe/CFP does not exhibit any further change, even under a high potential of 1.7 V_RHE_. This suggests that the as‐prepared Fe/CFP is already in the form of (oxy)hydroxide, which is widely regarded as an OER‐active structure. In Figure 3b, no clear change is observed in the Raman spectrum of Ni/CFP after 30 min immersion and at open circuit potential (OCP). However, as the potential increases, two new peaks at 475 and 553 cm^−1^ appear at 1.5 V_RHE_ and become more pronounced at higher potentials. These peaks correspond to the Ni^3+^—O vibration modes in NiOOH, indicating the phase transformation from NiO to NiOOH upon application of an external potential.^[^ ^31^ ^]^ Fe_1_Ni_1_/CFP exhibits a constant spectrum until 1.3 V_RHE_, as shown in Figure 3c. Two small shoulders located at 477 and 554 cm^−1^ emerge at a potential of 1.4 V_RHE_ and then increase significantly with increasing potential. Comparing Figure 3b,c, the formation of NiOOH is faster and in greater quantities in Fe_1_Ni_1_/CFP than in Ni/CFP. Notably, a broad and small hump ranging from 900 to 1200 cm^−1^ is observed in the spectra of Fe_1_Ni_1_/CFP at high potential, as shown in Figure 3d with a zoomed‐in view of this region. However, no clear signal is found in this region in the case of Ni/CFP. This signal can be attributed to active oxygen or superoxide species, which are widely reported as an active intermediate in alkaline OER catalyzed by CoOOH, Ni(OH)_2_, and NiFe hydroxide.[^30^, ^32^] This observation is consistent with the results from the pH‐dependance test. Decoupled proton–electron transfer promotes the formation of a highly active oxygen species in Fe_1_Ni_1_/CFP, resulting in improved kinetics and enhanced OER performance.^[^ ^33^ ^]^ After conducting in situ Raman experiments, the Ni/CFP and Fe_1_Ni_1_/CFP electrodes were thoroughly washed by dipping them in water multiple times and then dried at room temperature. The Raman spectra after drying are shown in Figure S8 (Supporting Information). The NiOOH in Ni/CFP transformed back into NiO after 1 day, while the NiOOH in Fe_1_Ni_1_/CFP could exist for 4 days due to the high population of NiOOH. Overall, the in situ Raman analysis reveals that Fe plays a significant role in the formation and stabilization of the active NiOOH, thereby boosting OER.

Our experimental findings confirm that incorporating iron into the catalyst significantly enhances OER performance, consistent with previous studies. In situ Raman spectroscopy reveals the formation of highly active oxyhydroxide species at elevated potentials, while Pourbaix slopes suggest modifications in the material's redox and acidic properties. However, the precise role of Fe remains unclear, and critical insights into the rate‐limiting steps and potential OER intermediates are still lacking. To address these gaps, we employ DFT calculations to investigate the OER reaction pathway on NiO and NiFeO models, leveraging a previously developed constant pH/potential simulation protocol.^[^ ^18^, ^19^ ^]^

All electronic structure calculations were performed at the PBE/def2‐SVP/CPCM(water) level of theory^[^ ^34^, ^35^, ^36^ ^]^ using the ORCA 5.0 program.^[^ ^37^, ^38^ ^]^ The core structural model employed in our simulations consists of a metal oxide cluster derived from the γ‐NiOOH phase, specifically incorporating the first coordination shell of Ni atoms around a central Ni atom, resulting in a Ni_7_O_24_ nanoparticle. The aqueous environment is represented using an implicit solvation model, while the effects of pH and potential are incorporated through explicit adjustments to charge and protonation states, in conjunction with an external proton and electron reservoir.^[^ ^18^, ^19^ ^]^ The pH/potential simulation protocol, detailed in recent publications, was conducted at pH 14 and a potential of 1.6 V. Notably, this model closely mirrors the properties of the synthesized catalyst, which exhibits nanosphere morphology, low crystallinity, and a high content of water and hydroxide species. For the simulation of the Fe‐modified material, in order to keep the model close to the 1:1 Fe/Ni ratio, we replace three Ni atoms with Fe, to obtain the Ni_4_Fe_3_O_24_ nanoparticle model. All possible permutations of the Fe atoms position are also considered, and the most stable structure is used for the OER profile calculation. Additional details of the structures and protocols are available in the Supporting Information (Figure S9 and S10, with atomic coordinates).

**Figure 4:**
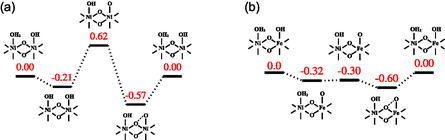
Reaction mechanisms and energies of intermediates (eV) for a) Ni_7_O_24_ and b) Ni_4_Fe_3_O_24_, and the corresponding OER energy profile for U = 1.6 V and pH 14 (PBE‐D3/def2‐SVP/CPCM (water) level of theory).

**Figure** **4**a illustrates the OER reaction profile for the Ni_7_O_24_ model. The *OH*OH intermediate exhibits a negative free energy (–0.21 eV), while the *O*OH intermediate lies at +0.62 eV, representing the rate‐limiting step in the reaction. Although the *OH*OH intermediate is easily accessed, further deprotonation and *O*OH intermediate generation is energetically less favorable. The M—OH group has a significantly higher pKa, leading to the reprotonation of the corresponding oxo species by adjacent OH_2_ groups. Moreover, at 1.6 V, oxidation of another Ni center fails to stabilize the *O*OH sufficiently. Figure 4b illustrates that the Fe‐modified material undergoes deprotonation of the *OH_2_*OH intermediate, leading to the formation of the *OH_2_*O intermediate, instead of the *OH*OH, where the oxo group is bound to the Fe center. *OH_2_*O and *OH*O intermediates are markedly stabilized, exhibiting a free energy of −0.32 and −0.30 eV, respectively. In the presence of Fe, further oxidation is achievable at 1.6 V, which induces the deprotonation of the OH group, a process not observed in the NiO case. These findings are consistent with the results of the electrochemical study, where the presence of Fe significantly alters the material's fundamental acidity and redox properties, thereby stabilizing reaction intermediates and facilitating rate‐determining steps.

## 3 Conclusion

In this study, we developed a rapid thermal treatment method for fabricating OER electrodes composed of nickel, iron, and CFP, eliminating the need for binders. The metal components are formed through the rapid thermal decomposition of precursors, yielding small, low crystallinity particles with high hydrous content. Protected by the carbon fiber network, these electrodes exhibit outstanding OER performance, with the Fe_1_Ni_1_/CFP catalyst demonstrating the highest catalytic activity and excellent stability.

In situ electrochemical Raman spectroscopy supported the rapid formation and good stability of oxyhydroxide and the occurrence of superoxide intermediate species on Fe_1_Ni_1_/CFP. To further elucidate the catalytic mechanism of FeNi‐based OER catalysts, electronic structure calculations were conducted under constant pH‐potential conditions. The results reveal that Fe directly influences OER catalysis by stabilizing oxo species and indirectly promotes the oxidation of adjacent Ni sites, facilitating deprotonation at these Ni centers. These theoretical insights align well with experimental findings, offering a deeper understanding of the synergistic effects of FeNi catalysts for the OER.

## Conflict of Interest

The authors declare no conflict of interest.

## Supporting information

Supplementary Material

## Data Availability

The data that support the findings of this study are available from the corresponding author upon reasonable request.
