# Supplementary material for: Experimental and Theoretical Insights into the Role of Iron in the Rapidly Fabricated Ni/Fe Electrodes for the Oxygen Evolution Reaction
Source: ChemSusChem. 2025 Jun 24;18(15):e202500281. doi: 10.1002/cssc.202500281 (PMC12302315; doi:10.1002/cssc.202500281)
Supplement: Supplementary file 1 — Supplementary Material [file CSSC-18-e202500281-s001.pdf]

Experimental and Theoretical Insights into the Role of Iron in the Rapidly Fabricated Ni/Fe Electrodes for the Oxygen Evolution Reaction

Yue Wang[a], Gustavo T. Feliciano[a], Ashwani Kumar[a], Alexander A. Auer[a] and Harun Tüysüz*[a],[b]

[a] Y. Wang, Dr. G. T. Feliciano, Dr. A. Kumar. Prof. Dr. A. A. Auer, Priv.-Doz. Dr. H. Tüysüz
Max-Planck-Institut für Kohlenforschung
Kaiser-Wilhelm-Platz 1, D-45470 Mülheim an der Ruhr, Germany
E-mail: tueysuez@kofo.mpg.de

[b] Prof. Dr. habil. H. Tüysüz
IMDEA Materials Institute
Calle Eric Kandel 2, Getafe, Madrid 28906, Spain
harun.tuysuz@imdea.org

Experimental Section

Chemicals: Carbon fiber paper (CFP, Toray Carbon paper, TGP-H-60, Alfa Aesar), Iron nitrate nonahydrate ($Fe(NO_3)_3 \cdot 9H_2O$; Sigma-Aldrich), Nickel nitrate hexahydrate ($Ni(NO_3)_2 \cdot 6H_2O$; Sigma-Aldrich), Ethanol (C_2H_5OH; Sigma-Aldrich, ≥99.9%), Potassium hydroxide (KOH, VWR Chemicals, 85.0 % assay) are ordered and used as they are.

Fabrication of electrode: The CFP was cut in the shape of a rectangular flag (a 1 cm x 1cm square with a 0.5 cm x 0.5 cm hilt). Metal precursors, $Fe(NO_3)_3 \cdot 9H_2O$ and $Ni(NO_3)_2 \cdot 6H_2O$, were dissolved in ethanol to prepare a 0.25 M solution with a desired Fe/Ni ratio. Then, 10 µl precursor solution was evenly dropped onto the 1 cm x 1cm square area of CFP, and dried with Ar flow for 30 s. Another 10 µl solution was dropped on the other side, resulting in a metal loading of 5 µmol on 1 cm x 1 cm CFP (Precursor/CFP). The muffin oven was preheated to 500°C at a ramping rate of 1000 K/h and then maintained at this temperature. The precursor/CFPs were promptly placed in the hot oven and the timer started immediately after closing the oven door. After specific durations (0.5 min, 1 min, and 3 min), the CFPs were swiftly removed. The samples are labeled accordingly as Fe/CFP, Ni/CFP, Fe_2Ni_1/CFP, Fe_1Ni_1/CFP, and Fe_1Ni_2/CFP.

Characterization: Transmission electron microscopy (TEM) images were collected with Hitachi H-7100 electron microscope. STEM images and corresponding elemental mapping were collected with Hitachi HD-2700 microscopes coupled with EDAX Octena T Ultra W 200mm^2 SSD windowless EDX detector. X-ray diffraction patterns are collected by a STOE theta/theta diffractometer with Cu $K\alpha_{1/2}$ radiation in the transmission mode. *Ex-situ* Raman spectroscopy measurements were conducted with an Invia Renishaw Raman microscope with a laser focusing on catalysts on carbon paper via a 50× objective lens. The laser source is 532 nm and max. 50 watts. 0.5 % laser intensity was used for *ex-situ* Raman. X-ray photoelectron spectroscopy (XPS) was conducted with a SPECS GmbH spectrometer and hemispherical analyzer (PHOIBOS 150 1D-DLD) under about 5 x 10^{-10} mbar with Al Kα X-ray source (E = 1486.6 eV). All XPS spectra were corrected with the C 1s peak of contaminant carbon at 284.5 eV.

Electrochemical measurement: All electrochemical measurements are conducted in a three-electrode system with potentiostat BioLogic VSP-300 with 85 % compensated IR drop. Pt wire was used as a counter electrode and reversed hydrogen electrode (RHE) as a reference electrode. The prepared electrodes were directly used as working electrodes. The tests were carried out in 250 ml 1M KOH electrolyte in a Teflon cell with a temperature of 25 °C, the electrolyte was purged with Ar gas for 30 min before the test. The linear sweep voltammetry (LSV) curves collected after 100 cyclic voltammetry (CV) circles were used to determine the activity. LSV curves were scanned from 0.7 V to 1.7 V vs. RHE with a scan rate of 10 mV/s, while CV curves were recorded from 0.7 V to 1.6 V vs. RHE with a scan rate of 50 mV/s. After LSV, impedance spectra were collected at 1.5 V vs. RHE with a frequency range from 105 Hz to 0.1 Hz. The stability test was conducted with chronopotentiometry (CP) mode fixing potential. *In-situ* electrochemical Raman spectroscopy was conducted with a homemade EC-Raman cell with quartz window in 0.1 M KOH. 5% laser intensity was used to get good resolution. First, the working electrodes were immersed in the electrolyte without applying any potential for 30 minutes to record the initial spectra after the solvation effect. Then, the In-situ Raman spectra were collected using chronoamperometry (CA) mode with a fixed potential.

Theoretical Section

Computational Details: All calculations are performed using the ORCA 5.0 program.[34, 36] Spin unrestricted density functional theory is employed as the electronic structure calculation method, using the generalized gradient Perdew-Burke-Ernzerhoff (GGA-PBE),[37] together with D3 dispersion correction with Becke-Johnson (BJ). The def2-SVP basis set is used, together with Def2/J auxiliary

basis with default grid.[38] A polarized continuum description of the environment around the catalyst model is employed, through the CPCM method.[39]

Catalyst Structural Model: The hexagonal nanoparticle model M_7O_{24} is employed throughout the study, as it was successfully previously used for modeling electrocatalytic properties of other transition metal oxides.[18] When modeling the $Ni_4Fe_3O_{24}$ mixed species, an additional simulation study was performed to choose the most stable configuration, regarding Fe spatial distribution, as presented in Figure S9. Only the most stable structure was considered for the evaluation of the OER reaction profile, with the respective intermediates.

pH/potential simulation protocol: From the chosen structural model, the pH/U protocol is applied.[18,19] The simulation is performed with several different total charge and protonation states, and the most stable structure at U=1.6V and pH 14 represents the resting state. Further OER reaction intermediates are derived, always from the previous intermediate, and reoptimized under the same pH/U condition, and are illustrated in Figure S10.

Atomic coordinates from the resting state structure of Ni_7O_{24} and $Ni_4Fe_3O_{24}$: Additionally, the atomic coordinates and total charge of the resting state structure of the studied materials, at U=1.6V and pH 14 are supplied.

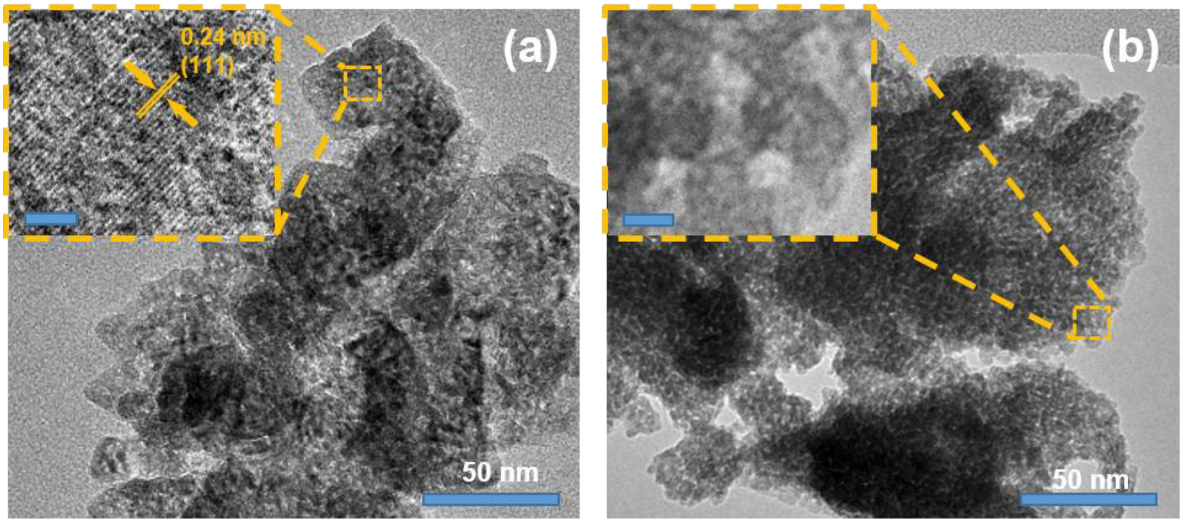

Figure S1. TEM image of (a) Ni/CFP and (b) Fe/CFP, insert figures are the corresponding HR-TEM images with 2 nm scale bar.

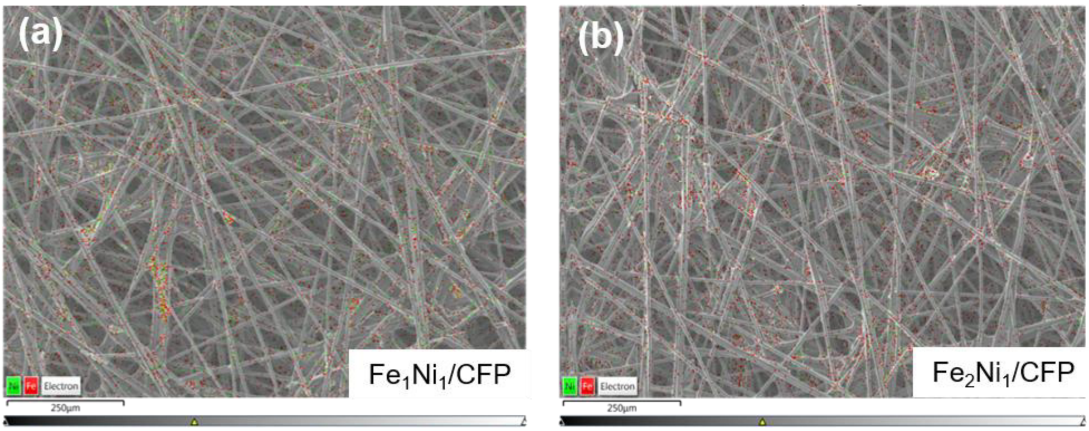

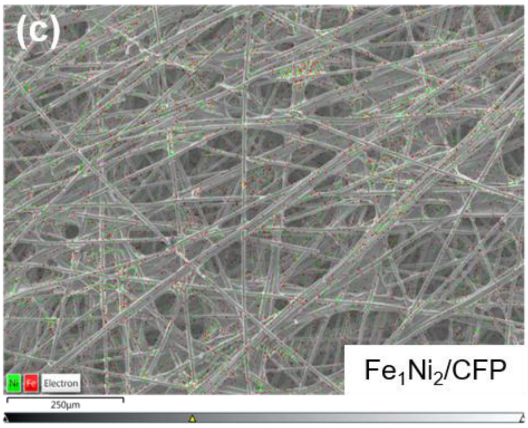

(d)

Sample	Ni (set as 1)	Fe
Fe_1Ni_1/CFP	1	1.00
Fe_2Ni_1/CFP	1	2.05
Fe_1Ni_2/CFP	1	0.49

Figure S2. SEM image of (a) Fe_1Ni_1/CFP, (b) Fe_2Ni_1/CFP, (c) Fe_1Ni_2/CFP and (d) corresponding Fe/Ni ratios.

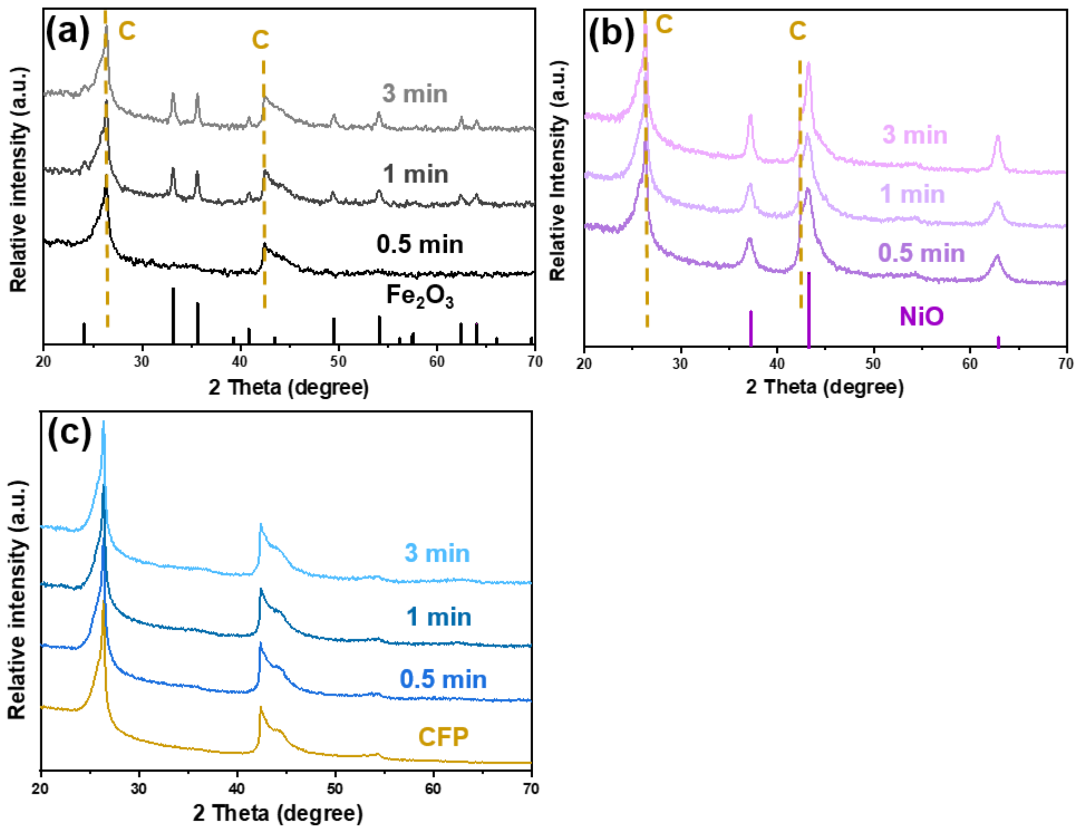

Figure S3. XRD patterns of electrodes (a) Fe/CFP, (b) Ni/CFP, and (c) Fe_1Ni_1/CFP after different thermal treatment times. The electrodes mentioned in the rest part are all heated for 0.5 min.

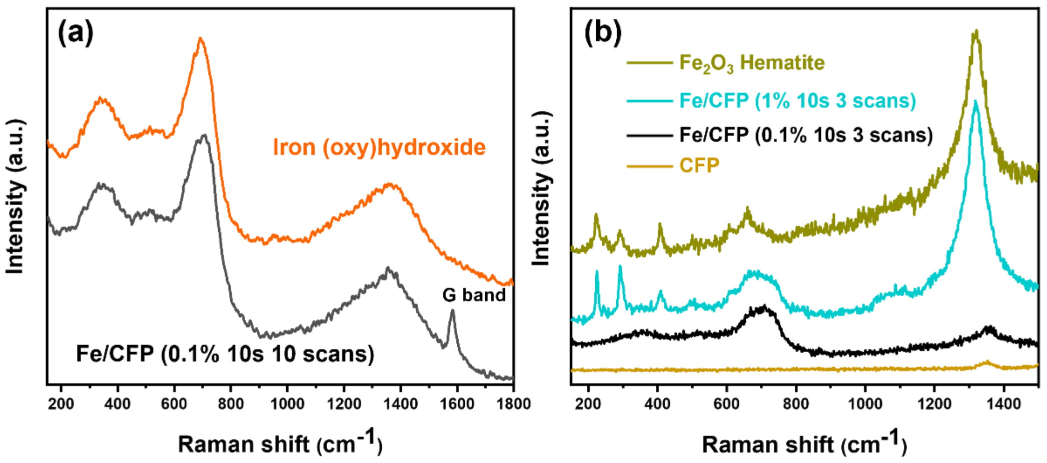

Figure S4. (a) Raman spectra of Fe/CFP and iron (oxy)hydroxide with 0.1 % laser intensity, 10s expose and 10 scans. (b) Raman spectra of Fe/CFP with different laser intensity and comparison with the spectra of Fe_2O_3 hematite.

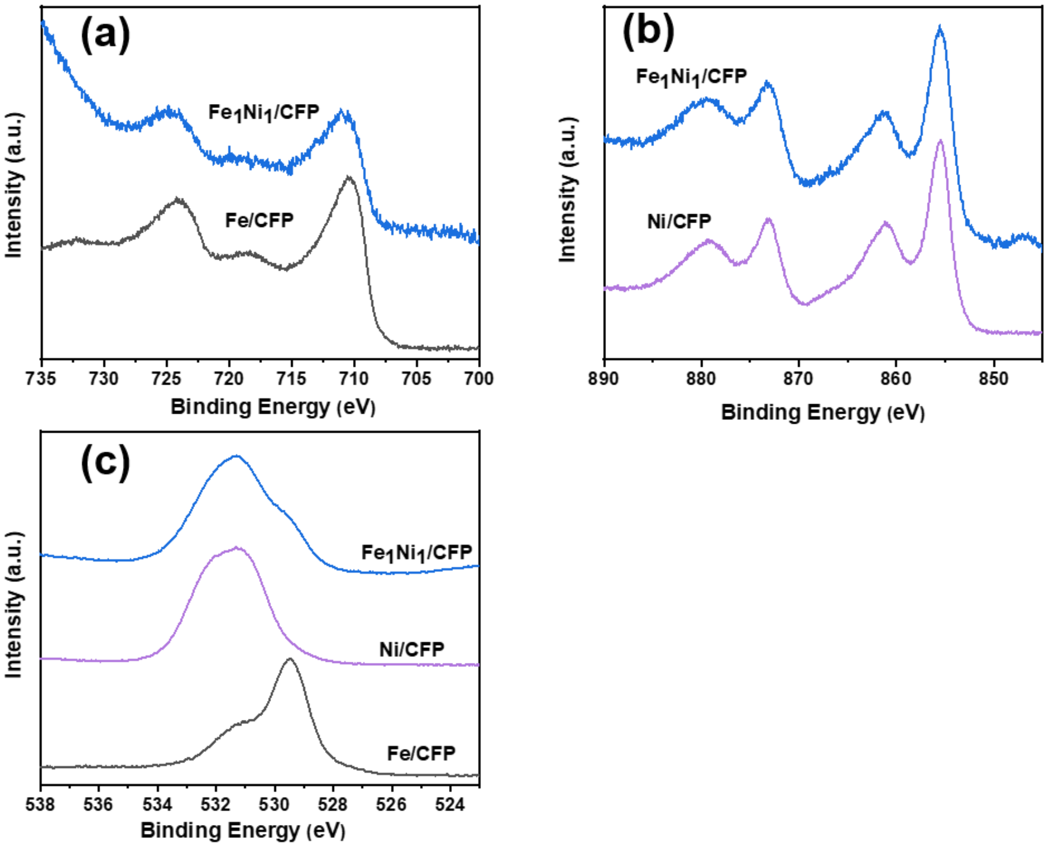

Figure S5. XPS spectra of (a) Fe2p in Fe/CFP and Fe_1Ni_1/CFP, (b) Ni2p in Ni/CFP and Fe_1Ni_1/CFP, and (c) O1s in Fe/CFP, Ni/CFP, and Fe_1Ni_1/CFP.

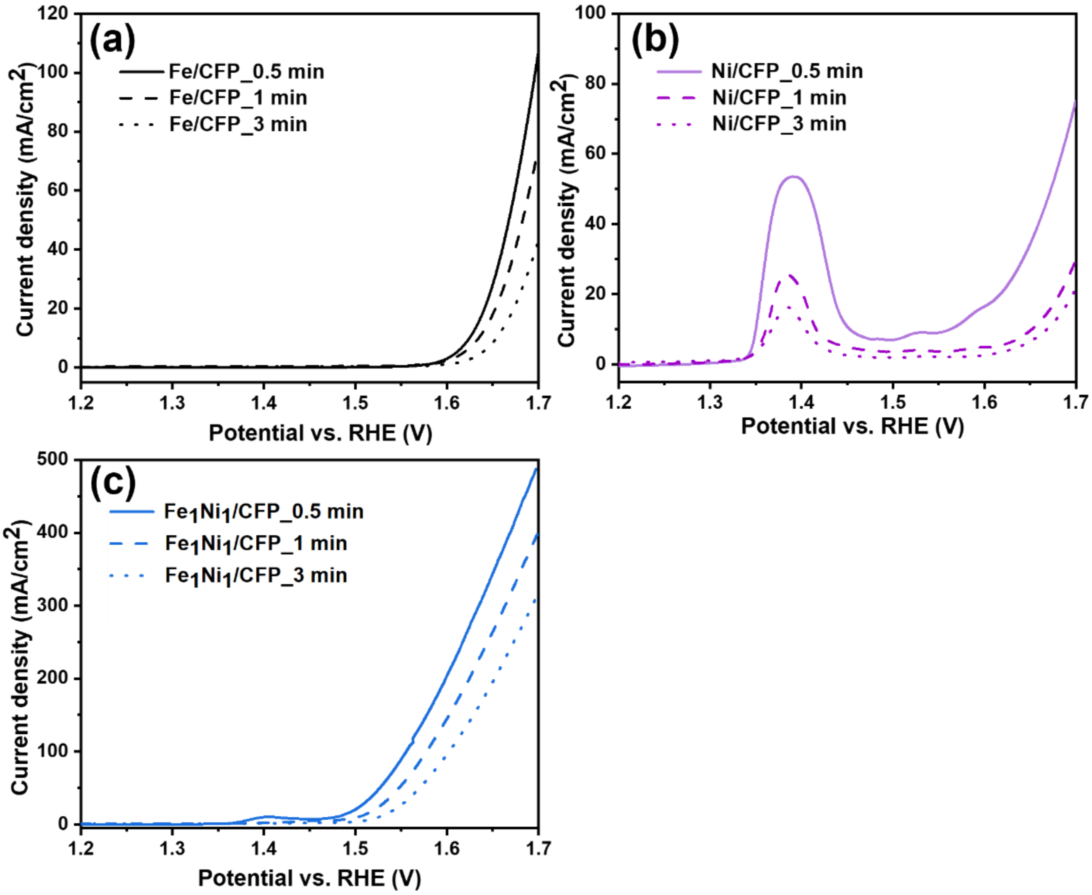

Figure S6. LSV curves of electrodes (a) Fe/CFP, (b) Ni/CFP, and (c) Fe_1Ni_1/CFP after different heating times. The electrodes mentioned in the rest part are all heated for 0.5 min.

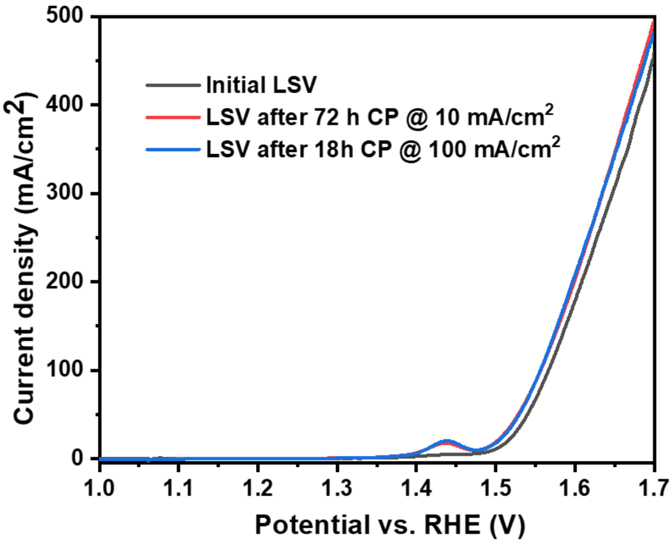

Figure S7. LSV curves of electrode Fe_1Ni_1/CFP before chronopotentiometry (CP) test, after 72 hours with current density at 10 mA/cm^2 and after 18 more hours with current density at 100 mA/cm^2.

Table S1. Activity comparison of Ni_1Fe_1/CFP with reported NiFe-based OER catalysts.

Catalyst	Electrolyte condition	η@10 mA/cm^2 (mV)	Tafel slope [mV dec^{-1}]	Reference
Ni_1Fe_1/CFP	1 M KOH	247	63	This work
Ni–Fe–OH/Ni_3S_2	1 M KOH	268	54	*Catal. Sci. Technol.* **2020**, 10, 1708
NiFe–LDH/NaMnO	1 M KOH	320	21	*Mater. Lett.* **2020**, 281, 128569
NiFeCr-6:2:1	1 M KOH	280	130	*Adv. Energy Mater.* **2018**, 8, 1703189
NiFe-LDH-Nanosheet	1 M KOH	197	100	*NanoRes.* 2019, 12, 1327
NiFe-LDH-Hollow nanoprism	1 M KOH	280	49.4	*Angew. Chem. Int. Ed. Engl.* **2018**, 57, 172
$Ni_{0.9}Fe_{0.1}$/NC	1 M KOH	330	45	*ACS Catal.* **2015**, 6,580.
$Ni_{70}Fe_{30}$/Cu foil	1 M KOH	260	55.6	*Inorg. Chem. Front.* **2017**, 4,1173.
Ni–Fe–OH/Ni_3S_2/Ni foam	1 M KOH	268	54	*Catal. Sci. Technol.* **2020**, 10,1708.
Fe_1Ni_1 oxide nanoparticle	1 M KOH	320	38	*Sci. Rep.* **2025**, 15, 8339
Co_xFe_yO-N/Ni foam	1 M KOH	304	52.7	*Sustain. Mater. Technol.* **2021**, 29, e00293

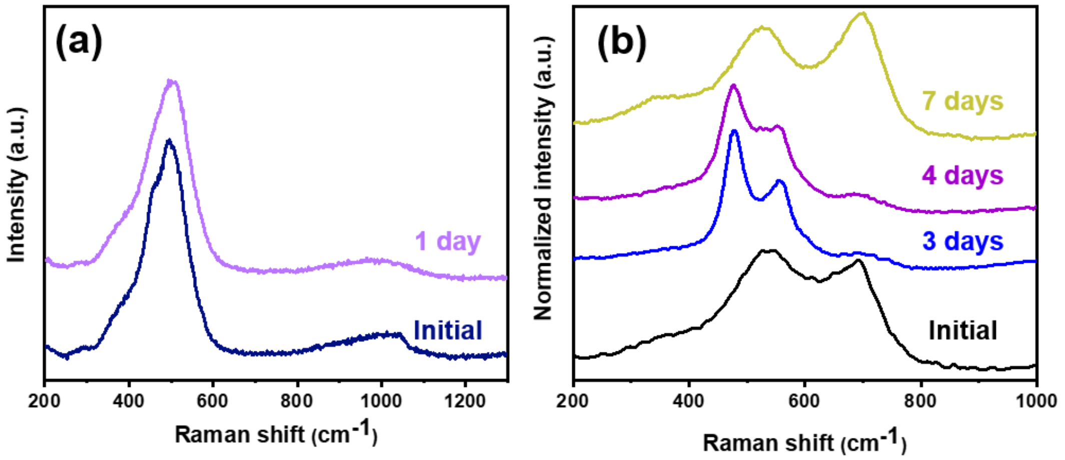

Figure S8. Raman spectra of as-prepared electrodes and dried ones after *in-situ* electrochemical Raman tests for (a) Ni/CFP and (b) Fe_1Ni_1/CFP.

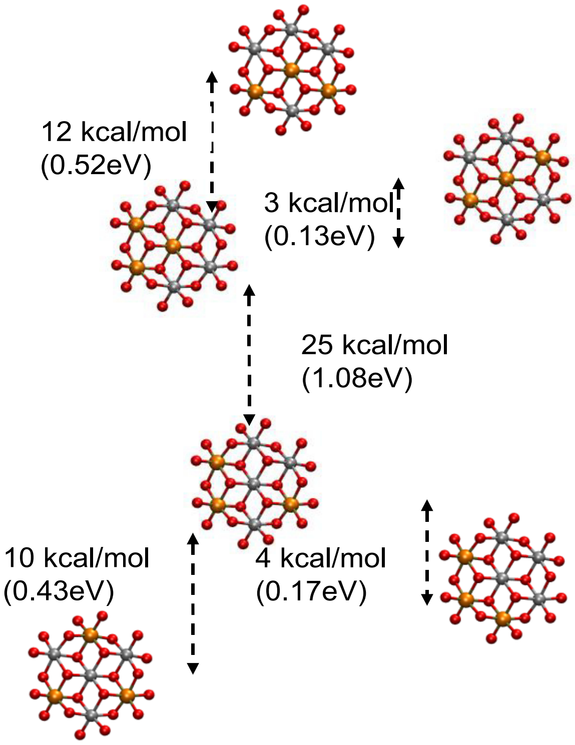

Figure S9. Relative stability of the possible configurations for $Ni_4Fe_3O_{24}$. Oxygen atoms are depicted in red, nickel in silver and iron in orange.

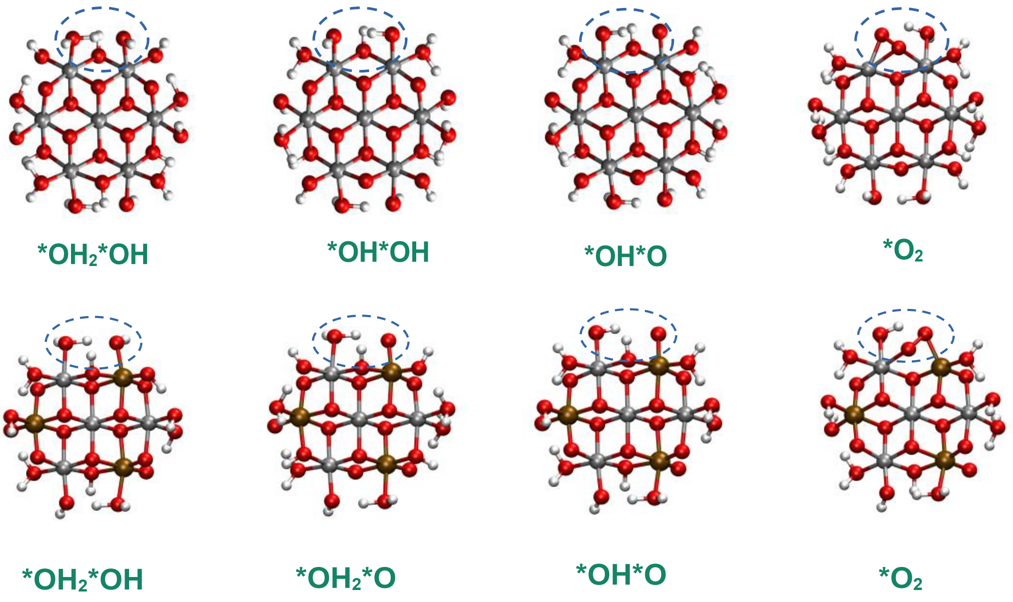

Figure S10. Structural model of OER reaction intermediates for Ni_7O_{24} and $Ni_4Fe_3O_{24}$ models at U=1.6V and pH 14.

Atomic coordinates for Ni_7O_{24} resting state at U=1.6V, pH 14:

53

Ni	2.74441318772566	4.38060720984850	12.81289721557722
Ni	2.65463831161247	7.21858703198966	12.84871396915672
Ni	7.58870840353054	4.51165458958168	12.95645392579518
Ni	7.49544980214500	7.38760564520734	13.02506315073077
Ni	5.15673048372543	3.05370799719189	12.81418514668152
Ni	5.12016336360417	5.86751028282531	12.85866284148791
Ni	4.98798072256799	8.66256465472018	12.90302280316643
O	1.17783731981269	4.49520247297352	13.93226266895766
O	1.12678169457665	7.02147371160208	13.91716032136928
O	6.02131633101369	1.78369243219008	13.88643863129231
O	5.93882224361347	4.43189432543054	13.81256624048864
O	5.81493761211092	7.30464263898572	13.89022786880400
O	5.78004357589263	9.96642354227665	14.00741779349688
O	3.64619884221712	3.17812249711638	13.86105863361022
O	3.49689227226350	5.80909976258536	13.78247941470039
O	3.48398834721812	8.45336244656297	13.92147651380525
O	8.20964699255980	3.04968830495845	14.08005956418879
O	8.12760411030895	6.09047816836745	14.22423174739767
O	8.00316111627310	8.85960231073749	14.16468905196141
O	4.24885601312601	1.75688165739797	11.71182091194336
O	4.33921161093783	4.45379008163177	11.83516839154495

O	4.27975942945347	7.26013070139231	11.87152186006819
O	4.02404348178444	9.92843179952079	11.81638980382829
O	9.28321265303451	4.76831256878273	12.05187227539966
O	9.15058927608560	7.32138243239598	12.14308707812441
O	2.07615004896034	2.96367174648990	11.76668635596211
O	1.99715821601800	5.79811809631819	11.70092199539969
O	1.92053430358200	8.62823866631030	11.83425168698472
O	6.69347209772730	3.15486310727651	11.66834601363061
O	6.82892225689393	5.95300615711215	12.01722534199228
O	6.61422637064839	8.59236821761186	11.81524560960430
H	4.62251998489506	1.89805103006836	10.81194340616501
H	4.37994213005579	9.81949347801066	10.90493553884135
H	9.19552672013569	4.45768233835613	11.12347053612919
H	8.96755624660631	7.66010266197837	11.23793397140170
H	1.43383786792862	4.14641608360130	14.81613015979325
H	1.38668447039631	7.39465922472925	14.78927869530577
H	5.55019701053574	1.85131934203587	14.74749783533301
H	7.06922982583239	9.44441807440042	14.17200973682803
H	5.32705961257688	9.85910044539634	14.87400450921126
H	1.01381797296098	5.77870423790898	11.73561553283400
H	3.01738958743346	9.42577168961908	11.78673190620874
H	3.24195526050459	2.21003156589855	11.67619573934411
H	8.91234646918237	2.54201245510640	13.61793817200407
H	9.32841656787642	5.82579530643666	11.99158308162825
H	9.10424886839421	6.21020509851334	14.27306817123354
H	1.08877547235251	5.56839035016745	14.04935945440460
H	1.30477359349605	9.06577943830489	12.46434563304433
H	7.34713844971122	2.42361363237765	14.08625915844053
H	1.51753497720859	2.44610892854122	12.38913279528504
H	7.07148067773928	9.46457960112979	11.83833928362065
H	7.16831836754745	2.29562570685288	11.72472190507873
H	8.68908494388563	9.39359118485165	13.70409519656478

Atomic coordinates for $Ni_4Fe_3O_{24}$ resting state at U=1.6V, pH 14:

49

Fe	2.72750653141739	4.23404624404294	12.80789447893877
Ni	2.66916097528017	7.20082175490558	12.91685830918374
Fe	7.63992979756798	4.40397203147076	12.93751777692635
Ni	7.46385804440344	7.33412031541494	13.00484269741722
Ni	5.21304066639168	3.05824163403625	12.90576308209102
Ni	5.11089766960843	5.84135874843396	12.92840278961219
Fe	5.03410734430441	8.70943798235685	12.95844072755278
O	1.11004482019680	4.47448898377989	13.94767735120362
O	1.15613512432283	7.01540908687210	14.02662560495789
O	6.03739800355022	1.79206461182286	13.99940125334624
O	5.93493065866605	4.45874089371757	13.91291136392914
O	5.87008305480195	7.26002252046481	13.94787079447502
O	5.79091661167713	9.90188403423586	14.16427537738909
O	3.56475413923297	3.18818450397041	13.88937426548557
O	3.48325828831663	5.81256027926292	13.84590148727348

O	3.54408154312020	8.52487953899691	13.91694076335167
O	8.25161436823548	3.20466872659821	14.07332915176117
O	8.20955667941167	5.93961541993534	14.09550444737474
O	8.10786937719465	8.79411891931305	14.15598726474482
O	4.36266172067783	1.73270680163325	11.72231872055234
O	4.38870292242471	4.44519885243221	11.89107052456161
O	4.28505062789798	7.24231462348923	11.95485382621702
O	4.23515323364836	9.86502725072504	11.75619734425953
O	9.16751864728968	4.76030454310768	11.91392187562966
O	9.07610013425689	7.28915048435206	11.92691150408090
O	2.14852429285276	3.10382435277619	11.72531044913408
O	2.02731505753578	5.81247848061744	11.76758512652859
O	1.94362609558675	8.66434213274731	11.79895506761219
O	6.86081908875277	3.29784548279700	11.90021202856226
O	6.75872648095532	5.93281421525814	12.01705509372472
O	6.56253004254923	8.60912463382653	12.00890767671449
H	4.74830824350128	1.86797520257258	10.82770308912486
H	4.77889578048662	9.83549047995579	10.93614000180081
H	8.83696309148585	7.64238634039515	11.04057868558445
H	1.31413875793242	4.11988976724649	14.83960364191045
H	5.67183565925906	1.99121309519870	14.89064646796935
H	7.22895877496402	9.34333570785606	14.28048534032818
H	5.24923947606136	9.86208584105780	14.98471553001628
H	1.04506901081223	5.82759375871646	11.73511157244325
H	2.80551298556829	9.22153187362644	11.64329603777991
H	3.40947000117680	2.13163610634657	11.64360848516742
H	7.49421486094749	2.50081167314617	14.09688505054217
H	9.19764959338624	6.23577453494914	11.79692975300817
H	9.18932982826608	6.02190262653123	14.12190514417444
H	1.05921809435551	5.53567107401583	14.06404700701623
H	1.39720423777692	9.25061286792572	12.36872938520851
H	0.36806402341974	7.32303031849949	13.52652068376174
H	9.05775819834336	4.33489820500698	11.03594706256784
H	8.67636946648397	9.37005414462881	13.59703659302999
